# Comparison of statistical methods for subnetwork detection in the integration of gene expression and protein interaction network

**DOI:** 10.1186/s12859-017-1567-2

**Published:** 2017-03-03

**Authors:** Hao He, Dongdong Lin, Jigang Zhang, Yu-ping Wang, Hong-wen Deng

**Affiliations:** 1 Department of Biostatistics and Bioinformatics, Center for Bioinformatics and Genomics, Tulane University School of Public Health and Tropical Medicine, 1440 Canal St., Suite 2001, New Orleans, LA 70112 USA; 2 Department of Biomedical Engineering, Tulane University, New Orleans, LA 70118 USA

**Keywords:** Active subnetworks, Protein–protein interaction, Searching algorithms

## Abstract

**Background:**

With the advancement of high-throughput technologies and enrichment of popular public databases, more and more research focuses of bioinformatics research have been on computational integration of network and gene expression profiles for extracting context-dependent active subnetworks. Many methods for subnetwork searching have been developed. Scoring and searching algorithms present a range of computational considerations and implementations. The primary goal of present study is to comprehensively evaluate the performance of different subnetwork detection methods. Eleven popular methods were selected for comprehensive comparison.

**Results:**

First, taking into account the dependence of genes given a protein-protein interaction (PPI) network, we simulated microarray gene expression data under case and control conditions. Then each method was applied to the simulated data for subnetwork identification. Second, a large microarray data set of prostate cancer was used to assess the practical performance of each method. Using both simulation studies and a real data application, we evaluated the performance of different methods in terms of recall and precision.

**Conclusions:**

*jActiveModules*, PinnacleZ and WMAXC performed well in identifying subnetwork with relative high precision and recall. BioNet performed very well only in precision. As none of methods outperformed other methods overall, users should choose an appropriate method based on the purposes of their studies.

## Background

In system biology, analysis of large biological networks has become major research topics in recent years. In order to better understand complex biological processes, diverse data sources revealing different aspect of biological functions are required for effective integration of knowledge. One of the most successful approaches has been to integrate protein–protein interaction (PPI) network with gene expression profiles to identify sets of genes and interactions that participate in a meaningful biological function, that is 'active subnetworks (modules)' [1]. Gene expression profiles monitor the transcription activities of thousands of genes simultaneously in various tissues and under diverse experimental conditions. PPI network, naturally complement interaction data primarily derived from experiments, provide a physical 'scaffold' with process-specific information that is correlated with cellular processes or disease states [2]. With the development of popular public databases, more and more research focuses of bioinformatics research have been to computational integration of network and gene expression profiles for extracting context-dependent active subnetworks.

Searching for active subnetworks has been a computationally difficult problem, known as Non-deterministic Polynomial-time (NP)-hard problem. Many methods for subnetwork searching have been developed, presenting a range of computational considerations and implementations. Different scoring functions have imposed scores on network nodes or edges or both [2, 3]. Besides, high-scoring nodes were prioritized as ‘seed genes’ for searching [4, 5]. Many searching algorithms, such as greedy searching, simulated annealing, genetic algorithms, have been proposed and applied to identify active subnetworks in recent studies. Because of the diversity of scoring functions and searching algorithms, it is impossible to obtain identical or similar subnetworks given the same input expression profiles and PPI network.

The primary goal of present study is to comprehensively evaluate the performance of different subnetwork detection methods. 11 methods were selected for comprehensive comparison. First, taking into account the dependence of genes given a PPI network, we simulated microarray gene expression data under case and control conditions. Then each method was applied to the simulated data for subnetwork identification. Second, an authoritative microarray data set of prostate cancer was used to assess and compare the performance of each method.

## Methods

### Subnetwork detection methods for comparison

As gene expression profiles can capture the transcription activities of thousands of genes simultaneously correlated with cellular or disease states, and PPI network provide a physical 'scaffold' with cell process-specific information, the integration of PPI network with gene expression profiles has become one of the most popular integrative approaches for extracting context-dependent active subnetworks. During the past decade various algorithms have been specifically developed to identify subnetworks in PPI network by integrating gene expression data and PPI network. 11 subnetwork detection methods were selected for comprehensive assessment based on the following rules. The input for each algorithm must be a network and an expression matrix or a list of seed genes or summary statistics based on the differential gene expression analysis. A brief summary of each method were given in the Table 1.

**Table 1:** Description of subnetwork detection methods

Method name	Algorithm	Tool type	Input
jAM_SA	Simulated annealing	Java;Cytoscape	PPI and *p*-values
jAM_GS	Greedy search	Java;Cytoscape	PPI and *p*-values
BioNet	integer-Linear Programming	R package	PPI and *p*-values
BMRF	Greedy search	Matlab	Gene expression matrix, PPI, label and seed genes
FEM	spin-glass algorithm	R package	PPI and t statistics
Cosine	Genetic algorithm	R package	Gene expression matrix and PPI
ClustEx	Clustering,shortest path	C	PPI and seed genes
WMAXC	Continuous genetic algorithm and a projection procedure	Matlab	Gene expression matrix and PPI
PinnacleZ	Greedy search	Java;Cytoscape	Gene expression matrix, PPI and label
KR	Klein-Ravi algorithm	Python	PPI, seed genes and scores of all nodes
Kwalk	Limited K-walks algorithm	Python	PPI, seed genes and scores of all nodes

*jActiveModules* is a plugin package in software Cytoscape. From a molecular interaction network it conducts searches of expression activated subnetworks [2], which show significant changes in expression over different conditions. The method combines a rigorous statistical measure for scoring subnetworks for finding subnetworks with high score through two different search algorithms: simulated annealing (jAM_SA) and greedy search (jAM_GS). The simulated annealing is aimed to search for the most highly scored subnetwork and the greedy search extends a subnetwork by adding one of its neighboring genes that maximizes a mutual information–based function. The input of *jActiveModules* is p-values of genes in the differential expression analysis between the two experimental conditions. For both search algorithms in *jActiveModule,* search depths from 1 to 3 were tested and maximum number of modules was set as 1.

*BioNet* is an R package for detection of functional modules through the integrative analysis of protein-protein interaction networks and gene expression profile. First, gene p-values calculated from the differential expression analysis are assigned to the nodes of the network. Second, based on gene p-values, scores are calculated by fitting a beta-uniform mixture model and then overall scores of network regions can be calculated. Third, subnetwork detection is modeled as a Prize-Collecting Steiner Tree (PCST) problem and an integer linear programming algorithm identifies the maximum scoring subnetwork [6, 7]. The *BioNet* allows for the fine tuning of the signal noise decomposition by the false discovery rate (FDR), therefore we scanned a range of FDRs to guarantee desirably sized modules and evaluated the obtained solutions in terms of recall and precision.

*Bagging Markov random field (BMRF)* approach is a BMRF-based method for subnetwork identification in the integration PPI data and microarray data with two different phenotypes (case/control). The BMRF approach integrates gene expression and PPI data based on on a framework of Markov random field modelling and maximum a posteriori estimation. It improves the subnetwork identification with a modified simulated annealing search algorithm and a so-called bagging aggregation scheme [8]. In BMRF, the parameter T, which controls the sharpness of the distribution of network score function, was set to 1 and the other parameter d distance was tested ranging from 1 to 3.

*Functional epigenetic modules (FEM)* algorithm is a functional supervised algorithm. It encapsulates the strength of associations of the genes with the phenotype in terms of the edge weights, in order to identify modules (subnetworks), where the edge weight density (called modularity) is significantly higher than in the rest of the network [9, 10]. An efficient spin-glass (SPG) module detection algorithm was used to identify modules, as it maximizes a relative weight density centered around specific seeds. The statistical significance of any inferred modules was assessed based on 1000 permutations, in which the node statistics were randomly permuted over the network followed by re-computation of the modularity values. One main tunable parameter (λ) in the FEM algorithm is used to determine the size of the inferred modules. We tuned λ to yield modules in a size range and evaluated them in terms of recall and precision.

*Condition specific sub-network (COSINE)* aims to identify a single optimal subnetwork of genes showing maximal alternation in terms of the expression pattern, given two or more microarray expression profiles under different conditions (case vs. control, etc.) [11]. In *COSINE*, a scoring function is used to jointly measure the differential expression of each gene (node) and gene-gene co-expression (edge). The parameter λ (0 ≤ λ ≤ 1) is a weight parameter to adjust for the size of the subnetwork in the PPI network. It uses the genetic algorithm to search for the single optimal subnetwork which maximizes the scoring function. A simple empirical procedure is used to select weight parameter λ, making it adaptive to the specific datasets being analyzed. As to the optimized choice of λ, we followed the procedure in the original paper [11].

*ClustEx* is a two-step method for identifying gene modules by integrating gene expression and PPI [4]. In the clustering step, the differentially expressed genes (DEGs) were clustered and partitioned into different groups by average linkage hierarchical clustering according to their distances in gene networks. In the extending step, the final gene modules were formed by adding intermediate genes on the k-shortest paths between the DE genes. It requires a seed gene set based on the DE analysis and the largest output cluster will be considered as the final subnetwork.

*Weighted maximum clique (WMAXC)* method aims to identify a condition-specific sub-network [12]. The weight of nodes and edges in a PPI network are calculated by scoring functions to measure differential expression of genes and gene-gene co-expressions for given two conditions. Then the maximal scoring subnetwork is identified by an optimization model. In WMAXC, A weight parameter λ is chosen to be adaptive to the dataset being analyzed and make the solution stable to the problem through a optimization model [12].

*PinnacleZ* is a Cytoscape plugin for classifying gene expression data by integrating gene expression and PPI network [13]. *PinnacleZ* requires three sources of input: a gene expression matrix, a class file and a PPI network. It scores subnetworks using the mutual information between aggregated gene Z-scores and sample labels. A greedy search is performed to find local subnetworks. In *PinnacleZ,* search depths from 1 to 3 were tested to provide a sufficient number of neighbors while keeping the search local.

*Klein-Ravi (KR)* algorithm will use a connected graph from seed genes as an initial tree [5, 14]. The iteration of the algorithm selects a non-tree node and a subset of at least two of the current trees to minimize the ratio called quotient cost. Once a node is selected, the shortest path is used to merge node and trees into one. For more details of the algorithm, please refer to the original paper [14].

*The limited K-walks (kwalk)* algorithm simulates random walks on a graph by the Markov Chain model [5, 15]. The relevance of an edge and a node in relation to the seed genes is evaluated by the expected times random walk passes starting from one seed to any of the others. A detailed elaboration can be found in the original paper [15].

## Results

### Simulation studies

PPI data was downloaded from the database of HPRD (Human Protein Reference Database, http://www.hprd.org/download), Release 9, 2010. To reduce the computational complexity, a subset PPI network containing 5,195 genes and 18,158 interactions was extracted from HPRD database. In this PPI network, 274 genes were randomly selected to be considered as the ground truth subnetwork of interactions. Given the PPI network and the ground truth subnetwork, gene expression data was simulated through two models considering the inter-dependence of genes in the network.

First, an MRF model was employed to determine the states of genes as ‘differentially expressed’ or ‘non-differentially (equally) expressed’ in the PPI network given the ground truth subnetwork. Let *X* be a binary vector indicating the states of genes in a PPI network G, 0 representing ‘equally expressed (EE)’ and 1 representing ‘differentially expressed (DE)’. The ground truth differential subnetwork was denoted as G_0_, which means *X*_{G0}_ = 1 and *X*_{G-G0}_ = 0. Then we can sample the gene state according to the following probability based on a Markov random field model: *p*_*i*_(*k*|⋅) ∝ exp(*γ*_*k*_ − *βμ*_*i*_(1 − *k*)), where *γ*_*k*_ and *β* were the parameters predefined, and *μ*_*i*_(1-*k*) denoted the number of neighbors of gene *i* having state 1-*k*, *k* = 0, 1. To introduce false positives in the sampled differential subnetwork, one more parameter (*w*) was added to control the probability of keeping initial states of ground truth DE genes and background EE genes. Here *μ*_*i*_(1-*k*) was defined as a function of parameter *w* as follows: ${\mu}_i\left(1- k\right)=\frac{w\cdot \left(1-{X}_i^{1- k}\right)+{\displaystyle \sum_{j\in {N}_i}\left(1-{X}_j^{1- k}\right)}}{w+{\displaystyle \sum_{j\in {N}_i}\left({X}_j^{1- k}+{X}_j^k\right)}},\kern0.5em \mathrm{where}\kern1em {X}^1= X,{X}^0=1- X.$ Here we chose *w* at *50* to generate simulation gene expression data set.

Second, based on the states of the genes, the gene expression levels were modeled in a Gamma-Gamma (GG) model, in which the observed variable *y* (gene expression level) follows a Gamma distribution having shape parameter *α* > 0 and scale parameter *β*_*g*_, with a mean value *μ*_*g*_ = *αβ*_*g*_. Mathematically, the probability density function of the GG model is defined by: $p\left( y\Big|\alpha, {\beta}_g\right)=\frac{y^{\alpha -1} \exp \left\{- y/{\beta}_g\right\}}{\beta_g^{\alpha}\varGamma \left(\alpha \right)}$. To finally generate simulation data, we fist sampled the scale parameter *β*_*g*_ based on Gamma distribution (*α*_*0*_ *, ν*) and then sampled gene expression levels using parameters (*α, β*_*g*_) given the states of genes. The parameters were the same as those in Newton et al. [16] (α = 10, α_0_ = 0.9 and ν = 0.5). Based on the differential state and gene dependency, the gene expression data were simulated with 50 samples in each phenotype (100 samples in total).

### Subnetwork identification performance assessment

As some methods do not prioritize genes in the subnetwork, we cannot use the area under the receiver operating characteristic (ROC) curve (AUC) as a criterion to evaluate the performance. Therefore, recall, precision and the combined *F*-measure were used to evaluate the performance of different methods. Precision and recall were defined as follows: Precision = (S_recovered_ ∩ S_ground_)/S_recovered_ = *TP*/*TP* + *FP*; Recall = (S_recovered_ ∩ S_ground_)/S_ground_ = *TP*/*TP* + *FN*, where true positive (TP) denotes the number of correctly identified genes, false positive (FP) denotes the number of falsely identified genes and false negative (FN) denotes the number of falsely unidentified genes. ${\mathrm{S}}_{\mathrm{recovered}}$ indicated the number of genes in the recovered subnetwork after applying each subnetwork identification method and ${\mathrm{S}}_{\mathrm{ground}}$ indicated the number of genes in the ground truth subnetwork. The traditional *F*-measure or balanced *F*-score (*F*_1_-score) is the harmonic mean of precision and recall, and can be used as a measure of total accuracy when equal importance is attached to recall and precision.

Performance comparisons of these methods were shown in Table 2. We can see that BioNet had the highest precision result, followed by jAM_GS and WMAXC. This is interesting because BioNet does not depend on a seed gene set but the significance of the gene expression. jAM_SA had the highest recall, followed by PinnacleZ, because jAM_SA used simulated annealing search. Different from other methods, ClustEx needed two-steps for subnetwork identification based on the assumption that a group of closely-connected differential genes were the signatures of the subnetworks. ClustEx had poorest performance among all the methods. Another poorest method is Cosine, possibly because it searched for the single optimal sub-network which jointly measures the changes of nodes as well as edges.

**Table 2:** Performance comparison on the simulated data for the subnetwork detection methods

Method	BioNet	jAM_GR	jAM_SA	Cosine	BMRF	WMAXC	FEM	PinnacleZ	KR	Kwalk	ClustEx
Precision	0.931	0.583	0.084	0.042	0.353	0.498	0.196	0.382	0.444	0.314	0.018
Recall	0.181	0.381	0.863	0.052	0.447	0.48	0.424	0.512	0.489	0.54	0.073
F-meausre	0.303	0.461	0.153	0.046	0.394	0.489	0.268	0.438	0.465	0.397	0.029

### Comparison on real data set with integration of PPIs

The performance of each method was tested using real data integrated with PPI network. Prostate cancer (PC) gene expression data was used (GSE3933), which contained the gene expression profiles of 71 prostate tumor and 41 normal samples [17]. Missing values in probes were imputed by the mean of observations and then data were standardized. Mean value of multiple probes mapped to a gene was computed as the expression level for that gene. Genes, which were contained both in PC gene expression profiles and HPRD PPI network, were included in the subsequent network analysis. After excluding self-interactions, there were 5335 genes (nodes) and 18234 interactions (edges). For the method evaluation, 703 genes related to PC from the Dragon Database of Genes associated with Prostate Cancer (DDPC) were used as reference genes [18]. This database comprehensively included genes all experimentally verified to be associated with PC. Among the 703 genes, 400 were included in the 5335 genes.

Fold enrichment was used to evaluate the performance of the methods and was calculated as (# of recovered genes)*5335/400*(# of selected genes), where selected genes were genes selected by the method, recovered genes were genes recovered by the method among the 400 reference genes, and 5335 represented the number of all genes in the entire network.

Table 3 showed the results of method comparison in real data set. The subnetwork size ranged from 196 to 1559. For example WMAXC extracted a subnetwork of the size 539 and resulted in a fold enrichment of 2.35. PinnacleZ had the highest fold enrichment of 2.49 with a subnetwork size of 246. It indicated that PinnacleZ outperformed all the other methods, although in the simulation data set, PinnacleZ had similar F-measure with jAM_GS and WMAXC.

**Table 3:** Performance comparison on prostate cancer gene expression data and PPI for the subnetwork detection methods

Method	BioNet	jAM_GS	jAM_SA	Cosine	BMRF	WMAXC	FEM	PinnacleZ	KR	Kwalk	ClustEx
Number of nodes selected	196	316	1559	243	601	539	233	246	328	466	419
Number of edges in the subnetwork	275	715	2987	102	1179	1698	292	503	472	771	495
Number of PC genes recovered	24	48	132	23	94	95	2	46	38	42	26
Fold enrichment	1.633	1.773	1.129	1.262	2.086	2.35	0.114	2.494	1.545	1.202	0.828

### Computational complexity and program usability

Apart from accuracy, another important attribute for each method is computational complexity. When dealing with a large high-dimensional data set, some methods may become unfeasible. BMRF and jAM_SA may cause such concerns. Most other methods would finish computing within reasonable time on standard desktop hardware even for large data sets. The usability of each method is determined by users’ familiarity with a particular platform (R, Matlab, JAVA and Python). *jActiveModules* and PinnacleZ are Cytoscape plugins, which offered notable user-friendly features with network analysis as well as convenient visualization functions. Similarly, BioNet will also provide analysis and visualization functions.

## Discussion

In the present study, we have performed a comprehensive assessment of various methods for subnetwork detection using simulation data and prostate cancer data. The key conclusion in this study can be summarized as follows.

First, although each of the methods was claimed to be effective in their original publications, based on the simulation scheme and read data sets they used. The subnetwork detection problem still needs further investigation. *jActiveModules*, PinnacleZ and WMAXC performed well in identifying subnetwork with relative high precision and recall. BioNet performed very well only in precision. As none of methods outperformed other methods overall, users should choose an appropriate method based on the purposes of their studies. Among the above four methods, for example, if only the summary statistics and p values are available in the study, BioNet will be a best choice of giving an exact functional module. If the study is interested in identifying subnetworks from selected genes/nodes, the best solution is *jActiveModules*, which provides options for searching from selected nodes and search depth. We suggest that investigators could use a combination of several different methods based on different principles. We suggest that a combination of BioNet, *jActiveModules* and PinnacleZ could be used.

Second, in terms of ease of use, some of the methods do not offer use-friendly interface or visualization functions for identified subnetworks. It is worth mentioning that the java plugins *jActiveModules* and PinnacleZ facilitate the analysis and visualization of subnetworks within an interface to Cytoscape.

Third, we found that the BMRF and jAM_SA were not applicable to large data sets, because of the computational complexity as well as the memory requirements for both methods increase greatly as the number of seeds increases.

Lastly, we suggest that interactome data (PPI) can be dissected and reorganized using high-level structures, such as pathways and GO terms. Those high-level structures can make sure that the output subnetworks are biologically meaningful and guide subnetwork detection methods to prune a global network without losing the important biological structures.

## Conclusion

In summary, the present study evaluated the performance of eleven different subnetwork detection methods. Using both simulation studies and a real data application, we evaluated the performance of different methods in terms of recall and precision. *jActiveModules*, PinnacleZ and WMAXC performed well in identifying subnetwork with relative high precision and recall. BioNet performed very well only in precision. As none of methods outperformed other methods overall, users should choose an appropriate method based on the purposes of their studies.

## Abbreviations

AUC: Area under the curve
BMRF: Bagging Markov random field
COSINE: Condition specific sub-network
DDPC: Dragon database of genes associated with prostate cancer
DEG: Differentially expressed genes
EE: Equally expressed
FDR: False discovery rate
FEM: Functional epigenetic modules
FN: False negative
FP: False positive
GG model: Gamma-Gamma model
HPRD: Human protein reference database
jAM_GS: Greedy search
jAM_SA: Simulated annealing
KR algorithm: Klein-ravi algorithm
Kwalk algorithm: Limited K-walks algorithm
PC: Prostate cancer
PCST: Prize-collecting steiner tree
PPI: Protein-protein interaction
ROC: Receiver operating characteristic
SPG: Spin-glass
TP: True positive
WMAXC: Weighted maximum clique
